# Procedural Sequence Learning in Attention Deficit Hyperactivity Disorder: A Meta-Analysis

**DOI:** 10.3389/fpsyg.2020.560064

**Published:** 2020-10-28

**Authors:** Teenu Sanjeevan, Robyn E. Cardy, Evdokia Anagnostou

**Affiliations:** ^1^Holland Bloorview Kids Rehabilitation Hospital, Toronto, ON, Canada; ^2^Department of Paediatrics, Medical Sciences Building, University of Toronto, Toronto, ON, Canada

**Keywords:** Attention Deficit Hyperactivity Disorder (ADHD), Procedural Deficit Hypothesis (PDH), sequence learning, serial reaction time task (SRT), meta-analysis

## Abstract

Previous literature proposes that the motor deficits in Attention Deficit Hyperactivity Disorder (ADHD) may be attributed to impairments of the procedural memory network, a long-term memory system involved in sensorimotor and cognitive skill development. A handful of studies have explored procedural sequence learning in ADHD, but findings have been inconsistent. A meta-analysis was conducted to begin to establish whether procedural sequence learning deficits exist in ADHD. The results of seven studies comprising 213 participants with ADHD and 257 participants with typical development (TD) generated an average standardized mean difference of 0.02 (CI_95_ −0.35, 0.39) that was not significant. Heterogeneity was significant across studies and could be partially attributed to the age of participants. We argue that procedural sequence learning appears to be preserved in ADHD and discuss potential explanations for and against this finding.

## Introduction

Attention Deficit Hyperactivity Disorder (ADHD) is one of the most commonly diagnosed neurodevelopmental disorders in children (Polanczyk et al., 2007; Rowland et al., 2015) with a prevalence rate of about 5% in school-aged children in Canada (Langlois et al., 2012). It is characterized by persistent inattention, impulsivity and hyperactivity, which can result in adverse social, educational, and health outcomes (American Psychiatric Association, 2013). ADHD seldom occurs without additional comorbid conditions (Brown, 2000; Gillberg et al., 2004). In fact, over half of children with ADHD also present with co-occurring motor impairment (Kaiser et al., 2015). Currently, the mechanisms underlying the motor impairment in ADHD are unknown. Previous literature suggests that deficits in procedural memory, a long-term memory system involved in the acquisition and consolidation of sensorimotor skills, may explain the co-occurring motor impairments in ADHD (Ullman, 2004). Specifically, this Procedural Deficit Hypothesis (PDH; Ullman and Pierpont, 2005) argues that motor sequence learning, supported by the procedural memory network, underlies the fine and gross motor impairments reported in some children with ADHD. A handful of studies have explored procedural sequence learning in ADHD, but findings across studies have been inconsistent. To address this gap in our understanding, we conducted a meta-analysis to begin to establish whether procedural sequence learning deficits exist in ADHD.

### Procedural Deficit Hypothesis

Procedural memory is an implicit long-term memory system that houses information on how to perform sensorimotor and cognitive skills such as driving a car or swimming. Although procedural memory is more commonly associated with the retention and near-automatization of learned skills, it also supports processes involved in the early stages of motor learning. These processes include (1) sequence learning: acquiring knowledge of the sequence of movements needed to perform a specific skill, (2) sequence planning: organizing the sequence of movements to perform the learned skill, (3) sequence execution: carrying out the planned sequence of movements of the learned skill, (4) adaptation: fine-tuning active movements to ensure the learned skill is correctly executed and, (5) consolidation: building and storing the motor memory that enables the automatized execution of the learned skill (Doyon, 2008; Doyon et al., 2009). Behavioral studies not only confirm the presence of motor impairment in ADHD, but also appear to suggest that learning a sequence of movements such as those needed to string blocks or alternate between hand and foot taps, may be specifically implicated in this disorder.

The neural structures that comprise the procedural memory network largely include frontal and basal ganglia circuits, the cerebellum and regions of the parietal and superior temporal cortices (Ullman, 2004; Mochizuki-Kawai, 2008; Doyon et al., 2009). Neuroimaging studies have consistently reported structural differences in some of these neural regions in ADHD relative to neurotypical development including the frontal cortex, basal ganglia and cerebellum (Mostofsky et al., 2002; Arnsten and Rubia, 2012; Cubillo et al., 2012; Stoodley, 2014).

Furthermore, Ullman (2001, 2004) proposed that the procedural memory network also supports language learning. Analogous to learning the sequence of movements needed to tie shoelaces, Ullman (2001, 2004) suggested that procedural memory also supports learning the sequence of rules and conditions needed to construct grammatical sentences. Previous literature has indicated that nearly two-thirds of children with ADHD present with co-occurring language disorders that impact language form including sound sequencing, word structuring and sentence formulation (Cohen, 1988; Johnson et al., 1999; Mueller and Tomblin, 2012; Sciberras et al., 2014).

Based on these findings collectively, Ullman and Pierpont (2005) hypothesized that neurodevelopmental disorders such as ADHD that exhibit sequence-based motor and language impairment as well as neuroanatomical differences in structures that comprise the procedural memory network, should also present with procedural sequence learning deficits.

### Procedural Sequence Learning in ADHD

To date, only a handful of studies have explored procedural sequence learning in ADHD. Most of these studies have used the serial reaction time (SRT) task (e.g., Karatekin et al., 2009; Vloet et al., 2010; Prehn-Kristensen et al., 2011; Laasonen et al., 2014; Weigard et al., 2016; Pedersen and Ohrmann, 2018), which is a button-press task commonly used to measure implicit visual-motor sequence learning (Nissen and Bullemer, 1987; Howard and Howard, 1997). For this task, a visual stimulus appears in one of four horizontally-arranged squares on a screen. Participants are asked to press one of four response buttons that corresponds to the location of the stimulus as quickly and as accurately as possible. Trials are organized into random and sequence blocks. For the random blocks, the location of the stimulus is arbitrary, but for the sequence blocks, the location of the stimulus follows a fixed sequence of locations that repeats several times within one block (e.g., 1-4-2-3-4-3-1-2 repeats 10 times). The length of the fixed sequence and the number of random and sequence blocks can vary.

Participants are not told about the fixed sequence. Despite having no explicit awareness of the repeating sequence, participants with typical development (TD) typically learn the sequence implicitly as indicated by (1) a significant reduction in response time between the first and last sequence blocks or, (2) a significant increase in response time in the random block immediately following the last sequence block on correct button-responses only. This change in response time is used as a measure of procedural sequence learning (Nissen and Bullemer, 1987).

Studies examining procedural sequence learning as measured by a reduction in response time have reported varied findings. Some studies have reported differences in procedural sequence learning in ADHD relative to TD (Karatekin et al., 2009; Prehn-Kristensen et al., 2011). One study by Karatekin et al. (2009) examined button press responses and oculomotor anticipation using an SRT task. Although response times decreased over time as expected, oculomotor responses indicated that children with ADHD were significantly slower at anticipating the next item in the sequence in comparison to children with TD. Karatekin et al. (2009) interpreted these findings as evidence for delayed sequence learning in ADHD. Another study by Prehn-Kristensen et al. (2011) explored the benefits of sleep on SRT performance. While children with ADHD and TD showed comparable reduction in response times across sequence trials, children with ADHD showed significantly greater reductions in response times after sleep relative to children with TD who showed no improvement after sleep. Prehn-Kristensen et al. (2011) concluded that the processes underlying procedural sequence learning may differ between ADHD and TD. Despite these findings, other studies have suggested that procedural sequence learning is unaffected in ADHD (Vloet et al., 2010; Laasonen et al., 2014; Weigard et al., 2016; Pedersen and Ohrmann, 2018). Specifically, these studies have reported comparable reductions in response times across sequences blocks on variations of the SRT task in children and adults with ADHD and TD.

Given the discrepancies across studies exploring procedural sequence learning in ADHD, it remains unclear whether procedural sequence learning deficits exist in ADHD. One explanation for these inconsistent findings is that most studies have defined, measured, and calculated procedural sequence learning differently. Another potential explanation is the low statistical power of some studies. Some studies had small sample sizes and therefore, may have been underpowered to detect small, but significant differences in procedural sequence learning in ADHD. Our last explanation is that procedural sequence learning may, in fact, not be impaired in ADHD.

### Study Objectives

To address this gap in the literature, we conducted a meta-analysis of SRT studies examining procedural sequence learning in ADHD to determine whether differences in procedural sequence learning are observed in this disorder. We specifically asked whether procedural sequence learning is significantly different in ADHD relative to TD using a single definition and calculation for procedural sequence learning. The findings of this meta-analysis are critical to inform our understanding of ADHD as a disorder, the PDH and future studies exploring the underlying cause of the motor deficits in ADHD.

## Methods

### Study Design and Selection

**Figure 1:**
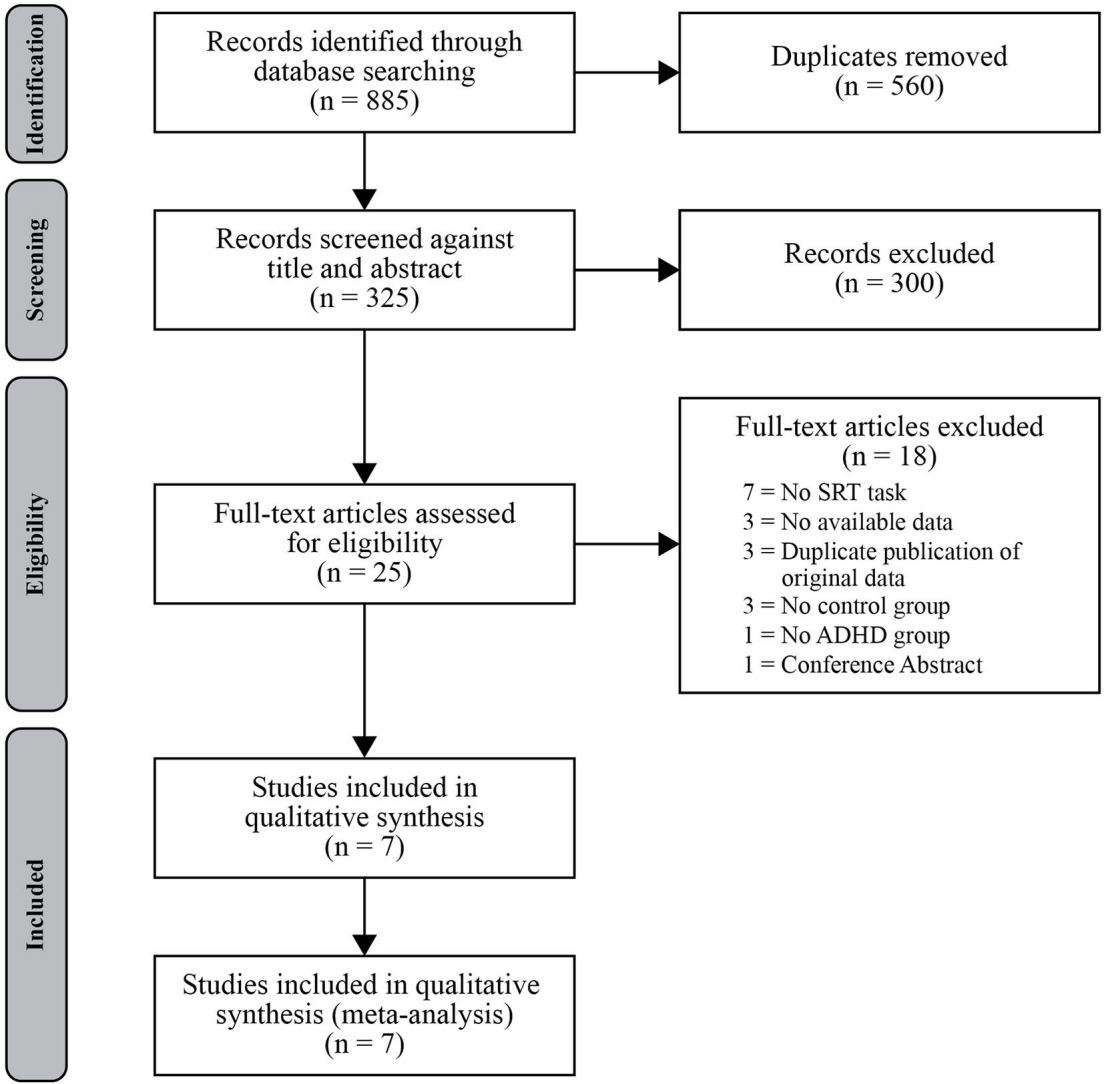
PRISMA flowchart showing the main stages of the literature search process.

A systematic search of the literature was conducted for journal articles and dissertations published on or before August 20, 2019. Databases used in this search included PsychINFO, Medline, and Embase (via OvidSP), CINAHL (via EbscoHost), Web of Science, Proquest, and the Cochrane Library. For database-specific search terms and methods for replication, see Appendix A in the Supplementary Material. The search strategy aimed to identify studies assessing procedural sequence learning using the SRT task in persons with ADHD, and at least one of the comparison groups had to consist of typically developing controls. Predetermined inclusion criteria also required the ADHD group to consist of individuals with a confirmed diagnosis of ADHD in accordance with DSM-IV or DSM-V criteria. Studies were ineligible if they did not have results available (e.g., ongoing clinical trials identified through Cochrane Library), as were articles that included data that had been previously published (e.g., duplicate publication of original data). Study selection was conducted and reported in accordance with the Preferred Reporting Items for Systematic Reviews and Meta-Analyses (PRISMA) Group guidelines (Figure 1) (Moher et al., 2009). Two authors (TS, REC) independently screened titles and abstracts, and both reviewed remaining full text articles for eligibility criteria. Where decisional conflicts arose, the reviewers deliberated until a consensus was reached. For each included study, reviewers recorded (when available) the mean age of the sample, sex distribution, IQ, source or basis of ADHD diagnosis, medication status, sequence learning task protocol, and performance measures (e.g., reaction time and accuracy).

### Analysis

To account for differences in the measure and calculation of procedural sequence learning across studies, we defined and measured procedural sequence learning on the SRT task as the mean difference in reaction time between the final random block and preceding sequence block as originally defined by the creators of the SRT task, Nissen and Bullemer (1987), and the most consistently used calculation in previous meta-analyses examining procedural sequence learning in neurodevelopmental disorders, including dyslexia, specific language impairment, and autism spectrum disorder (e.g., Lum et al., 2013, 2014; Obeid et al., 2016). If the mean difference in performance on the specified random and sequence blocks for a study's ADHD and TD groups was not directly reported, we calculated it from the published data or data provided by the authors using the general formula shown in (1) and (2). Larger mean difference values (D) represent a greater divergence of performance speed on the sequence and random blocks for a given group (specifically, faster performance on sequence blocks) and suggest greater procedural sequence learning.

(1) $$\begin{array}{l} \text{D}={\text{M}}_{\text{Sequence}}-{\text{M}}_{\text{Random}} \end{array}$$

(2) $$\begin{array}{l} \sigma_{\text{D}}=\sqrt{\frac{\sigma_{Sequence}^{2}}{n_{Sequence}}+\frac{\sigma_{Random}^{2}}{n_{Random}}} \end{array}$$

Where:

D = Raw mean difference in reaction times between final random block and preceding sequence block of the SRT task for a given group (ADHD or TD)
σ_D_ = Standard deviation of the mean difference (D)
σ^2^ = Standard deviation of the mean reaction time (M) for the random or sequence block
*n* = Number of subjects for the random or sequence block

Effect size data for each study was expressed as standardized mean difference (SMD) due to the variation in task methodology across studies (Higgins et al., 2019), using the mean difference and standard deviation calculations above, for ADHD and TD groups. SMD was calculated with a 95% confidence interval using the random effects model and analyzed using the inverse-variance method in Review Manager (RevMan) (2014) 5.3 software. SMD was calculated such that positive values indicated that the TD group exhibited greater sequence learning (greater difference in reaction time between the final random block and preceding sequence block), and negative values indicated greater sequence learning in the ADHD group. Effect size magnitudes were interpreted based on Cohen (1988), such that small, medium, and large effect sizes corresponded to SMD values 0.2, 0.5, and 0.8, respectively. Collected and extracted data used to compute the mean difference and effect size (SMD) is presented in Appendix B.

In order to determine the consistency of existing studies' findings, the *I*^2^ statistic was included in our analyses. The *I*^2^ is a useful index for quantifying inconsistency across a sample, as it describes the percentage of the variability within our sample due to heterogeneity rather than chance (Higgins et al., 2019). Classification for this index is typically low, medium, or high with values of 25, 50, and 75%, respectively. The methodological quality and risk of bias assessment was also conducted by each reviewer (TS, REC) independently, and any conflicts were resolved by discussion.

## Results

### Study Characteristics

The literature search identified 325 records after removing duplicates, and 25 articles remained after an initial title and abstract search for relevancy. Of these 25 articles assessed in full, 18 studies did not meet our predetermined inclusion criteria leaving seven studies that were included in our quantitative synthesis of the literature (Karatekin et al., 2009; Schnoll, 2009; Vloet et al., 2010; Prehn-Kristensen et al., 2011; Laasonen et al., 2014; Weigard et al., 2016; Pedersen and Ohrmann, 2018). No further articles were found through forward and backward reference searches of the included studies. Of those identified for inclusion in the meta-analysis, all but two studies (Laasonen et al., 2014; Pedersen and Ohrmann, 2018) investigated procedural sequence learning in children and adolescents with mean ages below 16 years of age for both the ADHD and TD groups. One study was a thesis project (Schnoll, 2009).

Table 1 outlines the summary characteristics of the studies included in the meta-analysis. The number of participants in each study ranged from 32 (Prehn-Kristensen et al., 2011) to 132 (Weigard et al., 2016), with a final dataset of 470 participants (ADHD = 213, TD = 257). Participants' IQ was at least within the normal range for six studies that had IQ eligibility criteria and/or reported means. For the two studies that did not report on IQ, Pedersen and Ohrmann (2018) instead assessed vocabulary using a German multiple-choice vocabulary test as a proxy measure of IQ while Schnoll (2009) provided no assessment of intellectual functioning. All but one study (Pedersen and Ohrmann, 2018) had predominantly male ADHD groups.

**Table 1:** Summary of studies included in meta-analysis.

**References**	**Sample size**			**Age (Mean ± SD)**	**IQ (Mean** **±** **SD)**		**Sex (% male)**
	**ADHD**	**TD**	**Total**				
Karatekin et al. (2009)	3	5	9	**ADHD** 12.8 (± 2.7) **TD** 12.8 (± 2.8)	**ADHD** 108 (± 14) **TD** 114 (± 13)	^‡^^†^^*^	**ADHD** 79% **TD** 45%
Laasonen et al. (2014)	2	3	5	**ADHD** 32.09 (± 8.71) **TD** 37.51 (± 11.14)	**ADHD** 102.55 (± 10.48) **TD** 109.91 (± 8.56)	^¤^	**ADHD** 64% **TD** 46%
Pedersen and Ohrmann (2018)	3	3	6	**ADHD** 31.5 (± 8.3) **TD** 28.2 (± 8.1)	**ADHD** – **TD** –		**ADHD** 47% **TD** 59%
Prehn-Kristensen et al. (2011)	1	1	3	**ADHD** 10.6 (± 0.88) **TD** 11.0 (± 0.99)	**ADHD** 106 (± 12.4) **TD** 109 (± 10.8)	^♢^	**ADHD** – **TD** –
Schnoll (2009)	2	2	4	**ADHD** 15.27 (± 0.03) **TD** 15.30 (± 0.31)	**ADHD** – **TD** –		**ADHD** 79% **TD** 60%
Vloet et al. (2010)	2	2	4	**ADHD** 14.3 (± 2.2) **TD** 14.0 (± 1.6)	**ADHD** 105 (± 8.2) **TD** 108 (9.5)	^‡^	**ADHD** 100% **TD** 100%
Weigard et al. (2016)	6	6	132	**ADHD** 9.96 (± 1.21) **TD** 10.18 (± 1.31)	**ADHD** 104.86 (± 12.16) **TD** 107.95 (± 10.66)	^†^	**ADHD** 59% **TD** 55%

*IQ was measured by the following assessments*.

‡ *Wechsler Intelligence Scale, 3rd ed. (WISC-III; Wechsler, 1991)*.

† *Wechsler Intelligence Scale for Children, 4th ed. (WISC-IV; Wechsler, 2003)*.

* *Wechsler Adult Intelligence Scale, 3rd ed. (WAIS-III; Wechsler, 1997)*.

¤ *Wechsler Abbreviated Scale of Intelligence (WASI; Wechsler, 1999)*.

♢ *Culture Fair Intelligence Test Revised Version (CFT-R; Weiss, 2006)*.

Across included studies, there was variation in the presentation of the SRT task and the operationalization of procedural sequence learning. In their assessment of procedural sequence learning in adults with dyslexia and ADHD, Laasonen et al. (2014) utilized a shape grammar-based structure for their SRT task as opposed to a simpler visual stimulus sequence, in order to make it more comparable with their other assessments. Pedersen and Ohrmann (2018) included a modified version of the SRT task that presented arrows as the visual stimulus and required participants to ignore the distracting information provided by the arrowhead direction in their responses. Instead of random trials, both Weigard et al. (2016) and Prehn-Kristensen et al. (2011) administered a new, untrained sequence following the sequence blocks in their studies. Prehn-Kristensen et al. (2011) also assessed the impact of sleep on procedural memory in children with ADHD and did not administer any random trials in their initial evening “learning” phase. Therefore, the sequence and random block data used in our analyses was collected from the subsequent daytime “retrieval” phase. The remaining three studies (Karatekin et al., 2009; Schnoll, 2009; Vloet et al., 2010) all followed a more traditional SRT paradigm as originally described by Nissen and Bullemer (1987).

### Evaluation of Publication Bias

**Figure 2:**
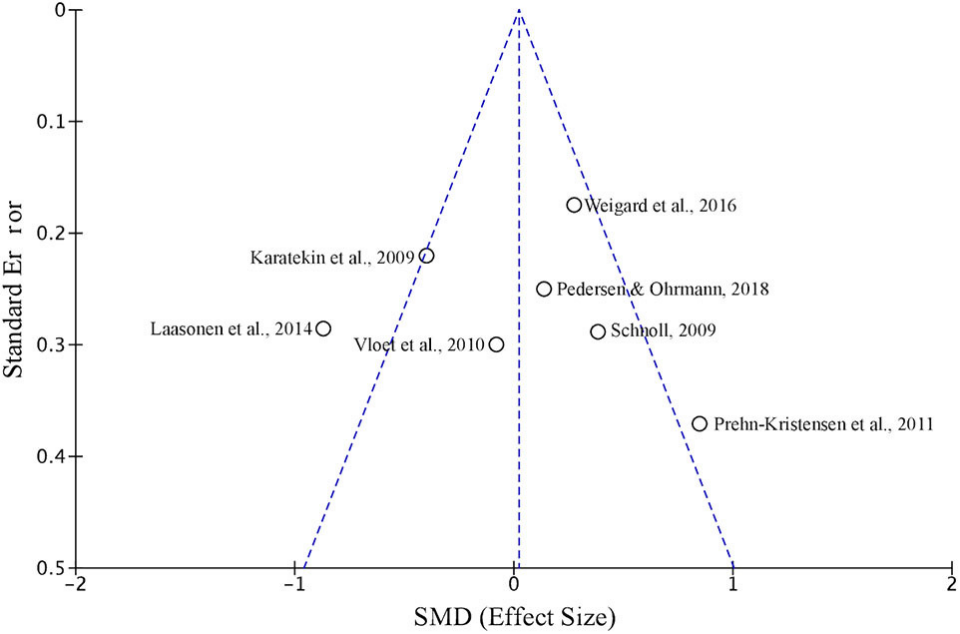
A funnel plot depicting SMD and standard error of the seven studies included in the meta-analysis of sequence learning in ADHD using the SRT task.

The funnel plot in Figure 2 illustrates potential publication bias, in which study level effect sizes and standard error are plotted around the weighted average effect size estimate. Evaluating publication bias in this manner dictates that if publication bias is present, the funnel plot ought to be skewed. In a visual examination of Figure 2, study level effect sizes are distributed relatively symmetrically around the weighted average, indicating that there is little evidence for publication bias in our selection. To quantify any possible publication bias, Egger's linear regression test of asymmetry was conducted (Egger et al., 1997). Egger's test was found to be non-significant [Intercept = 0.235, *t*_(5)_ = 0.067, *p* = 0.475], supporting the conclusion that bias was not found.

### Difference Between ADHD and TD on Procedural Sequence Learning Tasks

**Figure 3:**
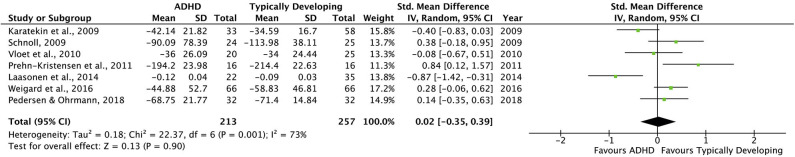
Forest Plot showing study weighted effect sizes (SMD) and overall effect size for all comparisons. Study level group means represent degree of sequence learning as the mean difference in RT between the final random block and preceding sequence block on the SRT task. Positive SMD values indicate greater sequnce learning in TD groups, whereas negative SMD values indicate greater sequence learning in ADHD groups.

A forest plot depicting study level and overall effect sizes with 95% confidence intervals is included in Figure 3. The meta-analysis showed that individuals with ADHD do not perform differently from TD on the SRT procedural sequence learning task [SMD 0.02 (−0.35 to 0.39)]. The heterogeneity of our study sample was moderate (*I*^2^ = 73%), indicating that much of the variability is due to heterogeneity rather than chance.

To explore the heterogeneity in our study, a single *post-hoc* subgroup analysis was conducted (Higgins et al., 2019). The subgroup analysis split studies into age-specific groups, to compare the overall effect of studies that involved only children and adolescents (5 studies) and only adults (2 studies). Subgroup meta-analyses revealed that the effect size remained small in the child and adolescent subgroup [SMD 0.16 (−0.22 to 0.55)], and the heterogeneity in this subgroup remained moderate (*I*^2^ = 65%). Results of the two studies, adults only subgroup revealed considerable heterogeneity (*I*^2^ = 86%) and a very wide, overlapping confidence interval [SMD −0.35 (−1.34 to 0.63)]. Of the two adult studies, Laasonen et al. (2014) depict a large effect size favoring procedural sequence learning in ADHD [SMD −0.87 (−1.42 to −0.31)], while the study by Pedersen and Ohrmann (2018) did not [SMD 0.14 (−0.35 to 0.63)].

## Discussion

This meta-analysis explored procedural sequence learning in ADHD, as indexed by performance on the SRT task. First, we aimed to determine whether there are significant differences in procedural sequence learning in ADHD relative to TD using a pre-defined measure of sequence learning. For the SRT task, procedural sequence learning was measured as the mean difference in reaction time between the final random block and preceding sequence block (e.g., Nissen and Bullemer, 1987; Lum et al., 2013, 2014; Obeid et al., 2016). Our results indicated that sequence learning was not significantly different between 213 participants with ADHD and 257 participants with TD compiled across seven studies, SMD 0.02 (−0.35, 0.39). Overall, these findings suggest that procedural sequence learning may be largely unimpaired in ADHD.

Our finding is consistent with several studies that have explored procedural sequence learning in ADHD (Vloet et al., 2010; Weigard et al., 2016; Pedersen and Ohrmann, 2018), reporting reductions in response time across sequence blocks in this disorder. There are, however, a subset of studies that have reported differences on SRT tasks, ranging from poor anticipation of the next item in the fixed sequence as indexed by slower shifts in eye gaze to gains in sequence learning after sleep in ADHD relative to TD (Karatekin et al., 2009; Prehn-Kristensen et al., 2011). Our analyses indicated that there was significant heterogeneity across studies, which may have led to the conflicting findings reported across studies. In addition to the way in which sequence learning was measured, we suspected that discrepancies across studies could also be attributed to differences in: (1) the length of the fixed sequence, (2) the number of exposures to the fixed sequence and, (3) the age of the participants, but have also found little evidence to support the potential influence of these task and participant characteristics. The following comparisons are based on the statistical findings of this meta-analysis, not the findings reported by studies themselves, unless otherwise stated.

First, we explored the length of the fixed sequence and its effect on performance on the SRT task. While many of the studies included in our meta-analysis used an 8-item sequence and did not generate performance differences in ADHD (e.g., Weigard et al., 2016; Pedersen and Ohrmann, 2018), differences were found when a 10-item sequence was used (e.g., Laasonen et al., 2014). Countering this argument, however, are studies such as Vloet et al. (2010), who also used a 10-item sequence and showed comparable sequence learning in ADHD and TD as well as studies such as Prehn-Kristensen et al. (2011), who used an 8-item sequence and found differences in sequence learning between ADHD and TD based on our calculation of sequence learning. It is, therefore, unlikely that the length of the fixed sequence can explain the significant differences reported by some ADHD studies.

Similar to the length of the fixed sequence, the number of exposures to the fixed sequence does not appear to contribute to differences in sequence learning in ADHD as well. While decreased exposure to the fixed sequence may have contributed to differences in sequence learning in ADHD (e.g., Prehn-Kristensen et al., 2011 exposed participants to an 8-item sequence 100 times), there are a few studies that have exposed participants to the fixed sequence far fewer times (e.g., Pedersen and Ohrmann, 2018 exposed participants to an 8-item sequence 25 times) and have reported preserved sequence learning in ADHD.

Third, the age of participants may have contributed to task performance differences in ADHD. Two of the eight studies included in this meta-analysis examined procedural sequence learning in adults with ADHD (Laasonen et al., 2014; Pedersen and Ohrmann, 2018), while the remaining five studies examined procedural sequence learning in children and adolescents with ADHD (Karatekin et al., 2009; Schnoll, 2009; Vloet et al., 2010; Prehn-Kristensen et al., 2011; Weigard et al., 2016). Among the adult ADHD studies, one study generated significant differences in procedural sequence learning based on our definition of procedural sequence learning (Laasonen et al., 2014), while the other did not yield differences (Pedersen and Ohrmann, 2018). Similarly, among the child and adolescent ADHD studies, one study generated significant differences in procedural sequence learning based on our definition of procedural sequence learning (Prehn-Kristensen et al., 2011), while the other four did not yield differences (Karatekin et al., 2009; Schnoll, 2009; Vloet et al., 2010; Prehn-Kristensen et al., 2011; Weigard et al., 2016). These findings suggest that age does not impact the procedural sequence learning abilities of individuals with ADHD differently than TD. This interpretation was supported by our *post-hoc* subgroup meta-analyses, which revealed no changes in the effect size or heterogeneity in the child and adolescent subgroup, but considerable heterogeneity and a wide, overlapping confidence interval for the two adult studies. These results, however, should be interpreted with caution given the low number of studies included in both subgroup analyses, particularly the adult subgroup. Overall, it appears that these suggested task and participant characteristics cannot adequately account for the significant group differences reported by some studies examining procedural sequence learning in ADHD.

### Alternative Explanations

Although our findings suggest that procedural sequence learning, as it has been operationalized in studies to date, may not be different in ADHD relative to TD, an alternative explanation could be that procedural sequence learning deficits could be attributed to inattention, a core deficit of ADHD. This interpretation suggests that if participants were not on attention-treating medications (e.g., methylphenidate) while performing the SRT task, we may expect to find differences in procedural sequence learning in ADHD such as those found in Karatekin et al. (2009) and Prehn-Kristensen et al.'s (2011) studies. However, all of the included studies that did not find significant group differences on the SRT task (e.g., Vloet et al., 2010; Weigard et al., 2016; Pedersen and Ohrmann, 2018) also reported that participants in their ADHD groups were medication-free for at least 24 h prior to participation in their studies. Therefore, stimulant medication does not likely explain the absence of procedural sequence learning deficits reported in this meta-analysis.

Another explanation may be that the declarative memory system plays a compensatory role for procedural memory dysfunction in ADHD. The declarative memory system is an explicit long-term memory system that forms, stores and retrieves fact-based information (Ullman, 2004), but also has associated roles in motor memory development and consolidation (Keisler and Shadmehr, 2010). Previous literature suggests that when procedural memory is impaired, the declarative memory system compensates for these inadequacies and supports processes such as sequence learning that are typically supported by the procedural memory network. This shift in dependence has been observed in specific language impairment (SLI), a neurodevelopmental disorder primarily characterized by deficits in language form and secondary non-linguistic deficits including motor impairment, with an unidentified cause (Ullman and Gopnik, 1999; Leonard, 2014). In a similar way, compensatory strategies involving the declarative memory system may occur in ADHD. Future studies should explore the role of declarative memory in ADHD to confirm this hypothesis.

A third explanation could be that other processes that underlie procedural motor learning such as sequence planning or visual-motor adaptation are instead impaired in ADHD and explain the motor impairments observed in this disorder. The fine and gross motor deficits reported in ADHD could potentially be attributed to poor sequential planning of the movements needed to perform specific motor tasks or perhaps weak abilities to modify ongoing movements to ensure the intended result is achieved. There is some evidence to support this speculation with at least one study reporting immature motor planning in ADHD as indicated by a delayed onset of movement given sufficient time as well as varied velocity profiles (Dahan and Reiner, 2017) and a few others reporting poor adaptation of hand and arm movements during reaching actions (Kurdziel et al., 2015) and on visuo-motor tracking tasks (Tirosh et al., 2006). Future research should explore whether performance on tasks that assess these motor processes are associated with the broader fine and gross motor deficits observed in ADHD.

Our final speculation is that procedural sequence learning may only be impaired in individuals with ADHD who also have comorbid motor impairment and is otherwise preserved in individuals with ADHD who do not have motor impairment. This interpretation suggests that procedural sequence learning deficits may be specific to motor impairment and may not be associated with ADHD. Given that none of the studies included in this meta-analysis describe the motor impairment status of their participants with ADHD, we cannot confirm whether the studies that reported differences comprised at least some participants with ADHD and comorbid motor impairment or whether the studies that did not report differences comprised a sample of participants with ADHD and no motor difficulties.

### Limitations

Although this meta-analysis grants us the statistical power to identify small effects in procedural sequence learning ADHD, if they exist, one caveat is that the studies may be biased in other ways (e.g., sample noise, researcher degrees of freedom) that were not explicitly outlined by the study and were therefore not captured or accounted for in this meta-analysis.

## Conclusions

We conducted a meta-analysis of seven studies examining procedural sequence learning as measured by a reduction in response time between the final random block and preceding sequence block in SRT tasks. Our results revealed no significant differences in sequence learning in ADHD relative to TD, which suggests that procedural sequence learning may be largely preserved in ADHD. It is, however, possible that if procedural sequence learning deficits exist, they may be compensated for by other memory networks or may only manifest in motor subtypes of ADHD. Alternatively, other procedural motor learning processes may instead contribute to the motor impairments in ADHD such as sequence planning and visuo-motor adaptation. We propose, as few others have before, that procedural sequence learning impairment is not a deficit of ADHD, but rather that of motor impairment and encourage future studies to explore this hypothesis further.

## Data Availability Statement

Requests to access these datasets should be directed to Teenu Sanjeevan, tsanjeevan@hollandbloorview.ca.

## Author Contributions

TS and RC: conceptualization, implementation, and writing. EA: conceptualization, supervision, and reviewing. All authors contributed to the article and approved the submitted version.

## Conflict of Interest

EA has received consultation fees from Roche and Quadrant, research funding from Roche, in-kind supports from AMO pharma, editorial Honoria from Wiley and book royalties from APPI and Springer. She holds a patent for the device, “Tully” (formerly Anxiety Meter). She has received royalties from APPI and Springer. The remaining authors declare that the research was conducted in the absence of any commercial or financial relationships that could be construed as a potential conflict of interest.
