# Supplementary material for: Procedural Sequence Learning in Attention Deficit Hyperactivity Disorder: A Meta-Analysis
Source: Front Psychol. 2020 Oct 28;11:560064. doi: 10.3389/fpsyg.2020.560064 (PMC7655644; doi:10.3389/fpsyg.2020.560064)
Supplement: Supplementary file 1 [file Table_1.pdf]

Appendix A

**Search strategy for CINAHL**	
S1	(TI attention deficit hyperactivity disorder*) OR (TI attention deficit disorder*) OR (TI ADHD) OR (TI ADDH) OR (AB attention deficit hyperactivity disorder*) OR (AB attention deficit disorder*) OR (AB ADHD) OR (AB ADDH) OR (attention deficit hyperactivity disorder*) OR (attention deficit disorder*) OR (ADHD) OR (ADDH)
S2	(TI procedural learning) OR (TI procedural memory) OR (TI implicit learning) OR (TI implicit memory) OR (TI implicit cognition) OR (TI implicit sequence learning) OR (TI statistical learning) OR (TI serial reaction time) OR (TI SRT) OR (TI ASRT) OR (AB procedural learning) OR (AB procedural memory) OR (AB implicit learning) OR (AB implicit memory) OR (AB implicit cognition) OR (AB implicit sequence learning) OR (AB statistical learning) OR (AB serial reaction time) OR (AB SRT) OR (AB ASRT)
S3	S1 and S2

**Search strategy for EMBASE/PsycINFO/Medline (via OVID)**	
S1	("attention deficit hyperactivity disorder*" or "attention deficit disorder*" or "ADHD" or "ADDH").ti,ab,kw.
S2	limit S1 to English language
S3	("procedural learning" or "procedural memory" or "implicit learning" or "implicit memory" or "implicit cognition" or "implicit sequence learning" or "statistical learning" or "serial reaction time" or "SRT" or "ASRT").ti,ab,kw.
S4	limit S3 to English language
S5	S2 and S4

**Search strategy for Web of Science**	
S1	TOPIC: (("procedural learning" or "procedural memory" or "implicit learning" or "implicit memory" or "implicit cognition" or "implicit sequence learning" or "statistical learning" or "serial reaction time" or "SRT" or "ASRT"))
S2	TOPIC: (("attention deficit hyperactivity disorder*" or "attention deficit disorder*" or "ADHD" or "ADDH"))
S3	S1 and S2

**Search strategy for Proquest**	
S1	ti,ab("attention deficit hyperactivity disorder*" OR "attention deficit disorder*" OR "ADHD" OR "ADDH")
S2	ti,ab("procedural learning" OR "procedural memory" OR "implicit learning" OR "implicit memory" OR "implicit cognition" OR "implicit sequence learning" OR "statistical learning" OR "serial reaction time" OR "SRT" OR "ASRT")
S3	S1 and S2

**Search strategy for Cochrane Library**	
S1	("attention deficit hyperactivity disorder*" or "attention deficit disorder*" or "ADHD" or "ADDH") in Title Abstract Keyword
S2	("procedural learning" or "procedural memory" or "implicit learning" or "implicit memory" or "implicit cognition" or "implicit sequence learning" or "statistical learning" or "serial reaction time" or "SRT" or "ASRT") in Title Abstract Keyword
S1	S2 and S3

Appendix B

Data Extracted from Studies

Study	Data extracted from study and/or authors	Values extracted	Data used to compute SMD in RevMan
Karatekin et al., 2010	Sample size, RT means and SD for Block 4 (final random block) and Block 3 (preceding sequence block) were extracted for ADHD and TD groups from data provided by author.	ADHD: M_S = 464.60 (± 84.86),<br>M_R = 506.73 (± 92.23)<br>TD: M_S = 421.37 (± 94.24),<br>M_R = 455.96 (± 85.44)<br>Sample size: ADHD = 33, TD = 58.	Calculated MD (± SD) between Block 3 and Block 4 and sample size for each group.<br>ADHD: MD = -42.14 (±21.82)<br>TD: MD = -34.59 (± 16.703)
Laasonen et al., 2014	Sample size, RT means and SD for Block 12 (final random block) and Block 11 (preceding sequence block) were extracted for ADHD and TD groups from data provided by author.	ADHD: M_S = 0.40 (± 0.11),<br>M_R = 0.52 (± 0.15)<br>TD: M_S = 0.46 (± 0.10),<br>M_R = 0.55 (± 0.13)<br>Sample size: ADHD = 22, TD = 35.	Calculated MD (± SD) between Block 11 and Block 12 and sample size for each group.<br>ADHD: MD = -0.12 (± 0.04)<br>TD: MD = -0.09 (± 0.03)
Pedersen & Ohrmann, 2018	Sample size, RT means and SD for Block 8 (final random block) and Block 7 (preceding sequence block) were extracted for each group from data provided by author.	ADHD: M_S = 326.53 (± 90.76),<br>M_R = 395.28 (± 83.26)<br>TD: M_S = 395.28 (± 83.26),<br>M_R = 0.55 (± 0.13)<br>Sample size: ADHD = 32, TD = 32.	Calculated MD (± SD) between Block 7 and Block 8 and sample size for each group.<br>ADHD: MD = -68.75 (± 21.77)<br>TD: MD = -71.40 (± 14.84)
Prehn-Kristensen et al., 2011	Sample size, RT means and SD for Block 6 (sequence block) and Block 7 (subsequent random block) during the retrieval phase were extracted for each group from data provided by author.	ADHD: M_S = 331.1 (± 79.4),<br>M_R = 525.3 (± 53.8)<br>TD: M_S = 274.0 (± 64.8)<br>M_R = 488.4 (± 63.2)<br>Sample size: ADHD = 16, TD = 16.	Calculated MD (± SD) between Block 6 and Block 7 and sample size for each group.<br>ADHD: MD = -194.20 (± 23.98)<br>TD: MD = -214.40 (22.63)
Schnoll, 2009	Sample size, RT means and SD for Block 5 (final random block) and Block 4 (preceding sequence block) were extracted for each group from published thesis.	ADHD: M_S = 423.22 (± 276.21),<br>M_R = 513.31 (± 266.83)<br>TD: M_S = 328.71 (± 131.82),<br>M_R = 442.69 (± 137.63)<br>Sample size: ADHD = 24, TD = 25.	Calculated MD (± SD) between Block 5 and Block 5 and sample size for each group.<br>ADHD: MD = -90.09 (± 78.39)<br>TD: MD = -113.98 (± 38.115)
Vloet et al., 2010	Sample size, RT means and SD for Block 4 (final random block) and Block 3 (preceding sequence block) were extracted for ADHD and TD groups from data provided by author.	ADHD: M_S = 513 (± 82),<br>M_R = 549 (± 83)<br>TD: M_S = 501 (± 98),<br>M_R = 535 (± 73)<br>Sample size: ADHD = 20, TD = 25.	Calculated MD (± SD) between Block 3 and Block 4 and sample size for each group.<br>ADHD: MD = -36.00 (± 26.09)<br>TD: MD = -34.00 (± 24.44)
Weigard et al., 2016	Sample size, RT means and SD for Block 6 (novel sequence block) and Block 5 (preceding original sequence block) were extracted for each group from published supplementary materials.	ADHD: M_S = 896.52 (± 280.7),<br>M_R = 941.4 (± 323.24)<br>TD: M_S = 832.03 (± 267.82),<br>M_R = 890.86 (± 269.97)<br>Sample size: ADHD = 66, TD = 66.	Calculated MD (± SD) between Block 5 and Block 6 and sample size for each group.<br>ADHD: MD = -44.88 (± 52.70)<br>TD: MD = -58.83 (± 46.81)
